# Optimized LL-37-Derived Peptides Exhibit Antitubercular Activity, Induce Membrane Disruption, and P-Type ATPase Transcriptional Responses in *Mycobacterium tuberculosis*

**DOI:** 10.3390/biom16050665

**Published:** 2026-04-30

**Authors:** Paola A. Santos, Milena Maya-Hoyos, Luz Mary Salazar, Claudia Andrea Cruz, Alver Cruz-Cacais, Mayerly Giraldo-Avila, Juliana Gómez-Manchego, Lineth Valentina Triana, Carlos Y. Soto

**Affiliations:** 1 Bioquímica y Biología Molecular de las Micobacterias―BBMM, Departamento de Química, Facultad de Ciencias, Universidad Nacional de Colombia, Carrera 30 N° 45-03, Bogotá 111321, Colombia; mmayah@unal.edu.co (M.M.-H.); lmsalazarpu@unal.edu.co (L.M.S.); alacruzca@unal.edu.co (A.C.-C.); mgiraldoav@unal.edu.co (M.G.-A.); jugomezma@unal.edu.co (J.G.-M.); 2 Relaciones Microbianas y Epidemiológicas Aplicadas al Laboratorio Clínico y Molecular—REMA, Facultad de Ciencias de la Salud, Universidad Colegio Mayor de Cundinamarca, Calle 28 N° 5B-02, Bogotá 110311, Colombia; candreacruz@universidadmayor.edu.co (C.A.C.); lvtriana@universidadmayor.edu.co (L.V.T.)

**Keywords:** *Mycobacterium tuberculosis*, antimicrobial peptides, LL37 analog peptides, P-type ATPases

## Abstract

Tuberculosis (TB), caused by *Mycobacterium tuberculosis* (*Mtb*), remains a major cause of morbidity and mortality worldwide, particularly due to the emergence of drug-resistant strains. Membrane-active antimicrobial peptides (AMPs) represent attractive therapeutic candidates because they target bacterial envelope integrity and disrupt essential cellular processes. We evaluated two rationally designed LL-37-derived peptides: a truncated C-terminally amidated analog (LL37-1) and a modified variant incorporating N-terminal acetylation and a single D-amino acid substitution (D-LL37). Dose–response analysis demonstrated that D-LL37 exhibited greater antimycobacterial potency, with lower inhibitory concentrations of 90% (IC_90_) and 50% (IC_50_) values (18.40 ± 0.39 μM and 10.11 ± 0.60 μM, respectively) compared with LL37-1 (25.44 ± 0.36 μM and 15.45 ± 1.40 μM). Fluorescence-based permeability assays revealed partial membrane disruption (36% and 44% at IC_90_ for LL37-1 and D-LL37, respectively), which was supported by ultrastructural alterations observed by scanning electron microscopy, including bacillary shortening, rough surface formation, cell clusters, and the presence of cellular debris, all of which are consistent with membrane damage. RT-qPCR analysis demonstrated significant upregulation of the P-type ATPase genes *ctpF*, *ctpA*, and *ctpH* following D-LL37 exposure. Collectively, these findings indicate that optimized LL-37-derived peptides exert antitubercular activity associated with envelope perturbation and coordinated activation of ion transport-related stress responses.

## 1. Introduction

Tuberculosis (TB), caused by *Mycobacterium tuberculosis* (*Mtb*), remains one of the leading causes of death from a single infectious agent worldwide. According to the World Health Organization (WHO), TB was responsible for approximately 1.25 million deaths in 2024, with nearly half a million cases associated with drug-resistant forms of the disease [1]. The emergence of multidrug-resistant (MDR) and extensively drug-resistant (XDR) *Mtb* strains has severely compromised current treatment regimens and underscores the urgent need for new therapeutic strategies with alternative mechanisms of action [2,3]. Researchers are investigating combination therapies that integrate novel antimicrobials with existing drugs. These therapies are being studied to enhance efficacy and reduce the emergence of resistance to anti-TB [3]. In addition, host-directed therapies (HDTs) offer a novel strategy for combating *Mtb* infections. These therapies modulate the host immune response to improve treatment outcomes [4].

Additionally, nanotechnology-based approaches are being explored. These approaches include the use of nanoparticles and nanocarrier-based delivery systems to enhance drug bioavailability and intracellular targeting [5,6]

Furthermore, approaches that identify non-canonical therapeutic targets for drug-resistant tuberculosis emphasize proteostasis (protein homeostasis) and adaptive transcriptional regulation as critical intervention points. Inhibitors targeting the ClpB protein, an essential disaggregase during cellular stress, and Rv0792c, a regulator of the oxidative stress response, have been identified. Inhibition of these proteins reduces bacterial persistence and enhances strategies against multidrug-resistant (MDR) and extensively drug-resistant (XDR) strains [7,8,9].

In this context, antimicrobial peptides (AMPs) have gained increasing attention as promising candidates for combating resistant infections. AMPs are evolutionarily conserved components of the innate immune system, present in all domains of life. They exhibit broad-spectrum antimicrobial activity against bacteria, fungi, viruses, and parasites, and many also display immunomodulatory properties [10,11]. Unlike conventional antibiotics, most AMPs primarily act by interacting with and disrupting cellular membranes, thereby permeabilizing and depolarizing them, and interfering with essential cellular processes. This membrane-targeting mechanism reduces the likelihood of rapid resistance development and makes AMPs particularly attractive as therapeutic scaffolds [12].

The mycobacterial cell envelope represents a complex and distinctive target for membrane-active compounds. It consists of a lipid-rich outer layer containing mycolic acids, arabinogalactan, and peptidoglycan, forming a highly impermeable barrier that contributes to intrinsic resistance to many antibiotics [13,14]. Despite its structural robustness, disruption of membrane integrity can compromise ion gradients, energy metabolism, and transport systems, thereby affecting bacterial viability. Therefore, peptides capable of perturbing mycobacterial membranes may exert antimicrobial effects not only through direct structural damage but also by triggering downstream cellular stress responses.

Among human AMPs, the cathelicidin LL-37 has been extensively studied for its antimicrobial and immunomodulatory properties. LL-37 displays activity against a variety of Gram-positive and Gram-negative bacteria. In addition, it has exhibited inhibitory effects on biofilm formation and bacterial adhesion [15,16]. However, the native peptide LL-37 is susceptible to proteolytic degradation and may exhibit suboptimal stability in physiological environments. To overcome these limitations, several structural modifications have been proposed, including truncation, amino acid substitutions, and the incorporation of D-amino acids to enhance protease resistance and improve antimicrobial potency [17]. Research on AMP in its D-enantiomeric form has demonstrated a wide therapeutic index, with enhanced activity compared to its L-enantiomeric counterparts [18].

Recent studies have shown that LL-37-derived peptides (AC-1, LL37-1, AC-2, and D-LL37) exhibit significant in vitro antifungal activity against various *Candida* species, including clinical isolates from vulvovaginal candidiasis. Among these peptides, LL37-1 and AC-2 demonstrated the lowest minimum inhibitory concentration (MIC) values [19]. In a separate study conducted by our group, LL-37 analogue peptides, particularly LL37-1 and D-LL37, displayed potent antibacterial activity against clinical isolates of *Staphylococcus aureus* and *Staphylococcus epidermidis*, effectively inhibiting both planktonic growth and biofilm formation, with D-LL37 exhibiting comparatively enhanced biological activity. [20]. Notably, D-LL37 contains a single D-amino acid substitution at the C-terminal region, a modification associated with increased structural stability and enhanced antimicrobial activity. In addition, we previously demonstrated that LLAP, another LL-37-derived peptide, inhibits plasma membrane ATPase activity in mycobacteria, suggesting that LL-37 analogues may affect membrane-associated enzymatic systems beyond simple physical disruption [21].

P-type ATPases are integral membrane proteins that use ATP hydrolysis to transport cations across biological membranes. In *Mtb*, several P-type ATPases are involved in the metal ion homeostasis and contribute to adaptation and virulence [22]. For example, CtpF is a Ca^2+^-transporting P-type ATPase necessary for full virulence and contributes to *Mtb* dormancy [23]. CtpA participates in copper transport and is involved in the response to redox stress [24]. CtpH is an ATPase that mediates calcium homeostasis and is necessary for *Mtb* to respond to stressful conditions [25].

In addition, over the past decade, our group has highlighted the relevance of P-type ATPases as virulence factors and potential therapeutic targets in *Mtb*. Genetic inactivation of the *ctpF* and *ctpA* genes leads to attenuation of *Mtb* in experimental models, underscoring their importance for bacterial survival under host-imposed stress [23,26]. Furthermore, structure-based approaches have identified small-molecule scaffolds targeting CtpF, reinforcing its potential as a drug target [27] Collectively, these findings support the hypothesis that membrane-active compounds may not only compromise envelope integrity but also modulate ion transport systems central to *Mtb* physiology.

Based on these considerations, we evaluated two bioinformatically optimized LL-37 derivatives, LL37-1 and D-LL37, for their inhibitory activity against *Mtb*, their effects on membrane permeability and ultrastructure, and their impact on the transcription of the *ctpF, ctpA*, and *ctpH* genes. By integrating functional assays, this study aims to provide mechanistic insight into how structural optimization of LL-37-derived peptides influences their antimycobacterial activity, their interactions with membrane-associated stress-response pathways, and the transcriptional modulation of P-type ATPase genes.

## 2. Materials and Methods

### 2.1. Bacterial Strain and Growth Conditions

All experiments were performed using the reference strain *Mtb*H37Ra (ATCC25177). Cultures were grown in Middlebrook 7H9 broth supplemented with 10% OADC (oleic acid 25 µg/mL, albumin 0.5% *w*/*v*, dextrose 0.2% *w*/*v*, and catalase 0.004% *w*/*v*), at 37 °C under constant agitation (80 rpm). Cultures were incubated for approximately 21 days until reaching the mid-logarithmic phase, corresponding to an optical density at 600 nm (OD_600_) of 0.6–0.8.

### 2.2. Peptide Design, Synthesis, and Preparation

LL-37-derived antimicrobial peptides were previously designed based on the central amphipathic region of the native human cathelicidin LL-37 sequence (FRKSKEKIGKEFKRIVQRIKDFLR) [20]. The truncated analog LL37-1 corresponds to a 23-residue derivative with the sequence GRKSAKIGKRAKRIVQRIKDFLR and was synthesized with a free N-terminal amino group and C-terminal amidation (–CONH_2_), the latter introduced to enhance structural stability and preserve overall charge. The modified analog D-LL37 incorporates two additional chemical modifications: (i) N-terminal acetylation and (ii) a single stereochemical substitution of phenylalanine at position 21 (L-Phe → D-Phe). C-terminal amidation was maintained in this variant. The introduction of a D-amino acid residue was intended to improve resistance to proteolytic degradation and modulate peptide–membrane interactions without altering net charge distribution.

Peptides were commercially synthesized and provided in lyophilized form by Biomatik Inc. (Ontario, Kitchener, ON, Canada). All peptides were supplied at a purity of >95%, as confirmed by the manufacturer via high-performance liquid chromatography (HPLC) and mass spectrometry analyses. Physicochemical specifications were provided in the supplier’s technical data sheet (Supplementary Materials). Lyophilized peptides were reconstituted in sterile deionized water to prepare stock solutions (1000 µM) according to their molecular weight. Stock solutions were aliquoted to avoid repeated freeze–thaw cycles and stored at −20 °C until use.

### 2.3. Determination of Inhibitory Concentrations of 50% (IC_50_) and 90% (IC_90_) Values by Dose–Response Analysis

An initial antimicrobial screening was performed using a resazurin-based microdilution assay to estimate inhibitory activity. This preliminary evaluation indicated inhibitory concentrations in the range of approximately 1–30 µM for both peptides. A refined dose–response analysis was conducted to determine IC_90_ and IC_50_ values. Peptide dilutions (1–30 µM) were prepared from 1000 µM stock solutions in 7H9–OADC–Tween 80 medium. Twenty-four concentration points were evaluated for each peptide to enable precise curve fitting. 100 μL of bacterial suspension (OD_600_ ≈ 0.04–0.06) in the 7H9–OADC–Tween 80 medium was separately mixed with 100 μL of serial peptide dilutions in 96-well flat-bottom microplates. Following 7 days of incubation at 37 °C with agitation (80 rpm), bacterial growth was quantified by measuring optical density at 595 nm using an iMark™ Microplate Absorbance Reader (Bio-Rad Laboratories, Inc., Hercules, CA, USA). Bacterial cultures supplemented with no peptide and 100 μg/mL gentamicin were designated as 100% (positive growth) and 0% (negative growth), respectively.

Dose–response curves were generated using nonlinear regression analysis (log[inhibitor] vs. normalized response, variable slope) in GraphPad Prism version 8.0.0 for Windows (GraphPad Software, San Diego, CA, USA, graphpad.com). From the fitted curves, IC_90_ and IC_50_ values were calculated. The experiment was performed in technical triplicate with two biological replicates.

### 2.4. Fluorescence-Based Assessment of Membrane Permeabilization

Membrane integrity following peptide exposure was evaluated using a dual-fluorescence viability assay (Cell-Check™ Viability/Cytotoxicity Kit for Bacterial Cells, ABP Biosciences, Rockville, MD, USA) according to the manufacturer’s instructions. This assay employs two fluorophores with differential membrane permeability: a green DNA-binding dye that labels total bacterial cells, and propidium iodide (PI), which selectively penetrates cells with compromised membrane integrity, emitting red fluorescence.

*Mtb*H37Ra cultures (OD_600_ ≈ 0.5–0.8) were divided into three aliquots, centrifuged at 10,000× *g* for 15 min, washed, and resuspended in 7H9-OADC medium. Suspensions were assigned to the following conditions: untreated control, LL37-1 (at the IC_90_), and D-LL37 (at the IC_90_). Samples were incubated for 2 h at room temperature with gentle agitation (80 rpm). After incubation, 100 μL of each suspension was transferred in triplicate to a black 96-well microplate. An equal volume (100 μL) of 2X staining solution containing the green fluorophore and PI was added to each well to obtain a final working concentration of 1X. The microplate was incubated at room temperature in the dark for 15 min. Fluorescence was measured using a Varioskan LUX multimode microplate reader (Thermo Fisher Scientific, Waltham, MA, USA) at excitation/emission wavelengths of 485/530 nm (green channel) and 485/630 nm (red channel). Positive (heat-killed bacteria) and negative (untreated viable bacteria) controls were included to validate staining performance and establish fluorescence. After fluorescence acquisition at excitation/emission, background fluorescence from medium-only wells was subtracted from all measurements. Membrane integrity was evaluated using the green/red fluorescence ratio (G/R), calculated as: G/R = Fgreen/Fred, where Fgreen corresponds to fluorescence intensity at 530 nm, and Fred corresponds to fluorescence intensity at 630 nm. To express membrane compromise as a percentage relative to untreated controls, the following normalization was applied: Membrane damage (%) = [1 − (G/R)_treated_/(G/R)_control_] × 100%. Untreated viable bacteria were defined as 0% membrane damage. All measurements were performed in technical triplicate for each of three independent biological replicates.

### 2.5. Scanning Electron Microscopy (SEM) for Evaluation of Peptide-Induced Morphological Alterations

SEM was performed to assess structural changes in *Mtb* following peptide treatment. *Mtb*H37Ra cultures (OD_600_ ≈ 0.8) were divided into three experimental conditions: untreated control, LL37-1–treated (at the IC_90_), and D-LL37–treated (at the IC_90_) samples. Bacterial suspensions were exposed to each peptide for 2 h at 37 °C under agitation (80 rpm).

Following treatment, cells were collected by centrifugation at 5000× *g* for 10 min and fixed in 2.5% (*v*/*v*) glutaraldehyde in phosphate buffer for 2 h at room temperature. Post-fixation was performed using 1% (*w*/*v*) osmium tetroxide for 1 h to preserve membrane-associated structures. Samples were subsequently washed three times with phosphate buffer. Dehydration was carried out through a graded ethanol series (30%, 50%, 70%, 90%, and 100%), with three final washes in absolute ethanol (15 min each). Ethanol was replaced with hexamethyldisilazane (HMDS) to promote chemical drying. Samples were mounted onto aluminum stubs using conductive carbon adhesive tape and sputter-coated with a thin layer of gold-palladium. Micrographs were obtained using a JEOL JSM-6460LV scanning electron microscope (JEOL Ltd., Tokyo, Japan) operated under standard high-vacuum conditions. Digital SEM micrographs were analyzed using ImageJ software, version 1.54j (National Institutes of Health, Bethesda, MD, USA).

SEM images were calibrated using the embedded microscope scale bar before measurement. Bacillary length was determined by manually tracing the longitudinal axis of clearly distinguishable individual cells using ImageJ software, version 1.54j (National Institutes of Health, Bethesda, MD, USA). Aggregated, overlapping, or partially visible bacilli were excluded from analysis to avoid measurement bias. For each experimental condition, at least 30 bacilli were measured from randomly selected, non-overlapping fields across three independent biological replicates. Measurements were performed using identical magnification and image acquisition parameters for all conditions.

### 2.6. Quantitative Real-Time PCR (qRT-PCR) Analysis of P-Type ATPase Transcriptional Response

To evaluate the transcriptional response of some P-type ATPase genes following membrane perturbation, total RNA was extracted from *Mtb*H37Ra cultures exposed to LL37-1 or D-LL37 under sublethal inhibitory conditions. Specifically, *Mtb*H37Ra culture (OD_600_ ≈ 0.8) was divided into three equal fractions, corresponding to experimental conditions: untreated control, LL37-1–treated (at the IC_50_), and D-LL37–treated (at the IC_50_) samples. Cells were harvested by centrifugation at 7600 rpm for 10 min at 4 °C, and pellets were washed three times with 7H9–OADC medium. The final pellet was resuspended in 7H9–OADC medium and exposed to LL37-1 or D-LL37 for 3 h at 37 °C.

Following treatment, cells were collected by centrifugation at 4 °C, washed twice with diethylpyrocarbonate (DEPC)-treated water, and processed for RNA extraction using TRIzol reagent (Invitrogen, Carlsbad, CA, USA) [28]. RNA was resuspended in DEPC-treated water, quantified using a NanoDrop™ OneC spectrophotometer (Thermo Fisher Scientific, Waltham, MA, USA), and its integrity was verified by electrophoresis on 2% agarose gels. To eliminate genomic DNA contamination, 2 μg of total RNA was treated with DNase I (EN0521, Thermo Scientific) in the presence of RNase inhibitor (Thermo Fisher Scientific) at 37 °C for 30 min. DNase was inactivated by adding 25 mM EDTA, followed by incubation at 65 °C for 10 min.

First-strand cDNA synthesis was performed using 2 μg of DNAse-treated RNA, dNTPs (10 mM), random primers (0.2 μg/μL), gene-specific reverse primers (0.02 mM), and OneScript^®^ Plus Reverse Transcriptase (Applied Biological Materials Inc., Richmond, BC, Canada), according to the manufacturer’s instructions. cDNA samples were stored at −20 °C until use.

Transcription levels of the genes 16SrRNA (housekeeping gene), *ctpF*, *ctpA*, and *ctpH* were measured using the Pfaffl method [29]. Measurements were carried out in triplicate, including a no-template control. qPCR assays were conducted using the SsoAdvanced Universal SYBR Green Supermix (Bio-Rad Laboratories, Inc., Hercules, CA, USA) on a CFX96 real-time PCR system (Bio-Rad Laboratories, Hercules, CA, USA). The cycling protocol consisted of an initial denaturation step at 95 °C for 5 min, followed by 39 cycles of 95 °C for 15 s, the primer annealing temperature (Tm) (°C) for 10 s, and 72 °C for 15 s.

## 3. Results

### 3.1. Dose–Response Analysis Reveals Enhanced Inhibitory Potency of D-LL37

**Figure 1:**
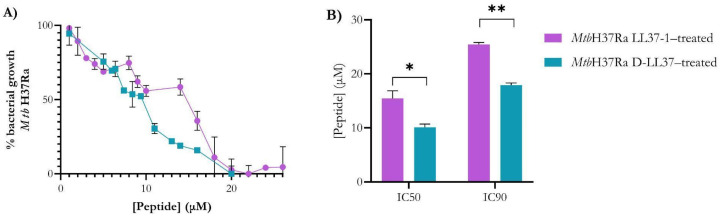
Dose–response curves of LL37-derived peptides against *Mtb*. (**A**) Nonlinear dose–response curves showing growth inhibition of *Mtb*H37Ra cultured in 7H9–OADC–Tween 80 medium in the presence of increasing concentrations of LL37-1 and D-LL37. OD_600_ of *Mtb* cells in the absence of peptides or in the presence of 100 μg/mL gentamicin was considered to represent 100% or 0% cell growth, respectively. (**B**) IC_50_ and IC_90_ values derived from curve fitting for both peptides were calculated using GraphPad Prism 8.0. Data represent the mean ± standard error of the mean from two independent biological replicates. Statistical significance between peptides was determined using Student’s *t*-test (* *p* < 0.05; ** *p* < 0.01).

Dose–response curves demonstrated concentration-dependent growth inhibition of *Mtb*H37Ra by both peptides. The sigmoidal profiles indicate a well-defined inhibitory transition within the micromolar range (Figure 1A). The IC_90_ values were 25.44 ± 0.36 µM for LL37-1 and 18.40 ± 0.39 µM for D-LL37. The lower IC_90_ of D-LL37 indicates that near-complete inhibition is achieved at a significantly lower concentration (*p* = 0.0026) than with LL37-1, confirming its superior inhibitory potency (Figure 1B). Similarly, the IC_50_ values were 15.45 ± 1.40 µM and 10.11 ± 0.60 µM for LL37-1 and D-LL37, respectively (*p* = 0.038) (Figure 1B). D-LL37 exhibited an approximate 28–35% reduction in both IC_50_ and IC_90_ compared with LL37-1. Overall, these results demonstrate that incorporation of the D-amino acid substitutions enhances antimycobacterial potency without substantially altering the overall dose–response pattern.

### 3.2. LL-37-Derived Peptides Induce Membrane Permeabilization in Mtb

**Figure 2:**
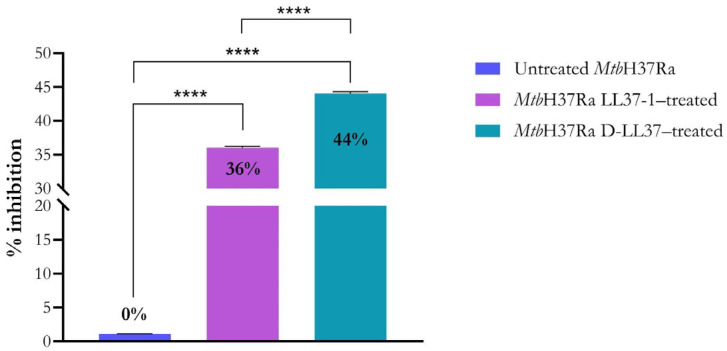
LL-37-derived peptides induce membrane permeability in *Mt*bH37Ra. The percentage of membrane damage was determined by the green/red fluorescence ratio in cultures treated with LL37-1 and D-LL37 at the IC_90_ concentrations. Untreated viable bacteria were defined as representing 0% membrane damage. Data represent the mean ± standard error of the mean from three independent biological replicates. Statistical significance was determined using a one-way ANOVA followed by Tukey’s multiple comparison test (**** *p* < 0.0001).

Membrane integrity was evaluated using a dual-fluorescence viability assay based on differential staining of intact and membrane-compromised cells. As shown in Figure 2, both LL-37-derived peptides induced significant membrane permeability alterations and therefore reduced cell viability in *Mtb*. Experiments were conducted at the IC_90_ values to ensure high-level growth inhibition while preserving sufficient cellular integrity for evaluation. Treatment with LL37-1 produced a 36% increase in membrane-compromised cells, whereas D-LL37 induced a 44% increase in membrane permeability relative to the untreated control (0%). These values indicate partial but significant envelope perturbation under highly inhibitory conditions. The greater permeability observed for D-LL37 is consistent with its enhanced inhibitory potency determined by dose–response analysis (Figure 1). Importantly, membrane disruption was incomplete, suggesting a controlled envelope destabilization rather than extensive lysis.

### 3.3. LL-37-Derived Peptides Induce Ultrastructural Alterations in Mtb

**Figure 3:**
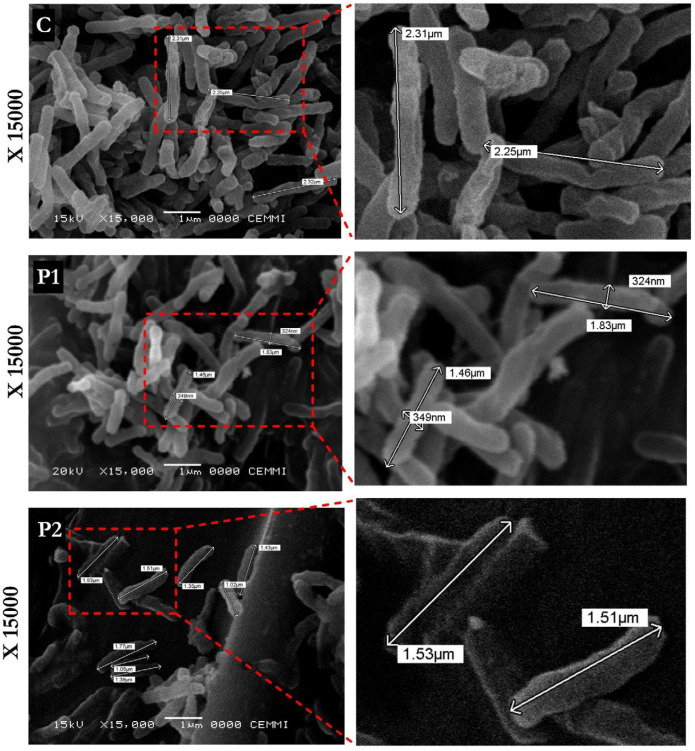
SEM analysis of *Mtb*H37Ra treated with LL-37-derived peptides. Representative micrographs (×15,000 magnification) of untreated control cells (C), LL37-1–treated (P1), and D-LL37–treated (P2) bacilli at their respective IC_90_ values for 2 h at 37 °C. Peptide-treated cells exhibit altered surface morphology, aggregation, and reduced bacillary length compared to controls. Scale bars are indicated in each panel.

SEM analysis revealed morphological alterations following peptide exposure compared to the untreated control (Figure 3). Untreated bacilli displayed the typical elongated rod-shaped morphology with smooth surfaces and a homogeneous distribution. In contrast, cells treated with LL37-1 or D-LL37 exhibited morphological changes characterized by a consistent reduction in bacillary length and possible alterations in cellular morphology. Subtle variations in surface appearance and cellular organization were observed in some fields, although these features were less pronounced and more variable compared to the consistent reduction in bacillary length. Overall, these observations support the occurrence of structural alterations associated with peptide exposure.

Morphometric analysis revealed that the control bacilli had an average length of 2.78 µm and an average width of 362.5 nm. Bacilli treated with LL37-1 exhibited a reduced average length of 1.65 µm and an average width of 352.6 nm. Similarly, D-LL37 treatment reduced the average bacillary length to 1.55 µm and the average width to 321.5 nm.

Overall, both peptides induced measurable reductions in cell dimensions and surface alterations compared to untreated controls, indicating structural perturbation of the mycobacterial envelope (Figure 3).

### 3.4. D-LL37 Induces Transcriptional Upregulation of P-Type ATPase Genes in Mtb

To evaluate whether exposure to antimicrobial peptides affects the transcription of P-type ATPase genes, the expression levels of *ctpF*, *ctpA*, and *ctpH* were quantified by qPCR following treatment of *Mtb*H37Ra with LL37-1 and D-LL37 at their respective IC_50_ values. Sublethal inhibitory concentrations were considered more appropriate to ensure preservation of cellular integrity and RNA quality while enabling detection of adaptive stress responses.

**Figure 4:**
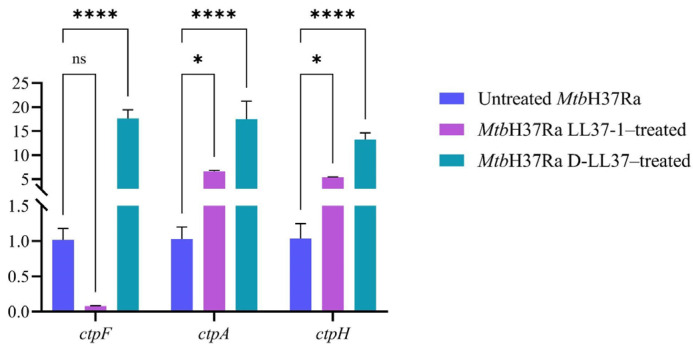
Relative expression of genes encoding P-type ATPases in *Mtb*H37Ra following LL37-1 and D-LL37 exposure at IC_50_ concentrations. Expression levels were normalized to a constitutive gene (*16Sr*RNA). Transcription levels are expressed as the ratio between peptide-treated and untreated *Mtb*H37Ra cells (control; transcription ratio ≈ 1.00). Data represent the mean ± standard deviation from four technical replicates. Statistical significance was determined using a two-way ANOVA followed by Holm–Šídák’s multiple comparisons test (*ns p* > 0.05, * *p* < 0.05; **** *p* < 0.0001). The untreated sample was used as the control condition. Statistical analyses were performed using GraphPad Prism version 9 (GraphPad Software, San Diego, CA, USA).

As shown in Figure 4, exposure to D-LL37 significantly increased the expression of all analyzed P-type ATPases genes compared to untreated *Mtb*H37Ra cells (**** p < 0.001).

Treatment with LL37-1 resulted in a statistically significant increase in *ctpA* and *ctpH* expression (* *p* = 0.0144 and * *p* = 0.0498, respectively), while no statistically significant change was observed for *ctpF* expression (*ns,* *p* = 0.6543), despite a marked decrease in fold change (≈0.08), suggesting a strong biological trend that did not reach statistical significance under the experimental conditions. The study’s findings indicated a correlation between membrane perturbation or general stresses caused by the peptides’ activity and the changes in transcription levels of *ctpF*, *ctpA*, and *ctpH*, rather than an effect on ATPase activity.

## 4. Discussion

AMPs have emerged as promising alternatives to conventional antibiotics due to their ability to target bacterial membranes and disrupt essential cellular processes, often with a reduced propensity for resistance development [12]. Among them, the human cathelicidin LL-37 has attracted particular interest because of its broad-spectrum antimicrobial activity [30,31]. However, the clinical application of native LL-37 is often limited by its susceptibility to proteolysis and its potential for host–cell toxicity [32].

Consequently, structural optimization of LL-37-derived sequences has become a rational strategy to enhance antimicrobial efficacy while preserving selectivity and safety [32,33]. In this study, LL-37-derived peptides inhibited *Mtb*H37Ra growth in the micromolar range, with the D-LL37 exhibiting greater potency than LL37-1. Functional assays demonstrated that this inhibitory effect is associated with partial membrane permeabilization, indicating moderate alterations in envelope integrity rather than extensive membrane disruption.

The antimycobacterial activity of LL37 derivatives can be understood in terms of net charge, membrane interaction, and peptide secondary structure. AMPs typically adopt α-helical conformations, which allow them to interact effectively with lipid interfaces and hydrophobic regions. This interaction often results in membrane perturbation [34]. Structural optimization strategies suggest that increasing helical content correlates with stronger insertion into lipid bilayers, thereby improving antimicrobial potency [35].

Previous circular dichroism analyses of LL37-derived peptides, including LL37-1 and D-LL37, showed a predominant α-helical conformation under membrane-mimetic conditions. These results and in silico predictions support their amphipathic character and a strong folding propensity [20]. Enhanced helical propensity under membrane-mimetic conditions is correlated with improved antimicrobial activity. Although these biophysical characterizations were not performed in the current study, mechanistic interpretations about membrane insertion, structural stability, and stereochemistry are informed by previous evidence and relevant literature. The antimicrobial activity observed in this study is directly supported by susceptibility assays, whereas mechanistic explanations are inferred from established similarities to previous findings rather than from direct measurements.

In both LL37-derived peptides, the substitution pattern (F^1^→G, KE^5−6^→AK, and EF^10−11^→RA) increases positive charge and favors α-helical conformations. For example, Gly at position 1, despite being a helix breaker in some contexts, is at the N-terminus, where it may influence N-terminal flexibility and local α-helical structure [36]. Furthermore, the substitution of negatively charged glutamate with positively charged arginine and lysine residues enhances electrostatic interactions with the phospholipids and glycolipids of the mycobacterial membrane and increases the stability of the α-helix conformation necessary for effective membrane engagement [37,38].

Additionally, the post-translational mimetic modifications, such as C-terminal amidation in LL37-1 and combined N-terminal acetylation with C-terminal amidation in D-LL37, are well known to enhance helical stability and protease resistance [39,40]. Moreover, the introduction of a D-Phe at position 21 in D-LL37 does not alter the net charge, but it may reduce proteolytic cleavage and promote α-helical structure during interactions with lipid interfaces [41].

Conversely, a crucial aspect to validate the structural optimization of these AMPs is the balance between antibacterial activity and toxicity. The previously reported data showed that the hemolytic and cytotoxic data of D-LL37 exhibited a remarkably favorable activity-toxicity balance [20]. In fact, the IC_90_ value (18.40 µM) remains below the established cytotoxicity threshold for mammalian cells (<25% cytotoxicity up to 20 µM) and is only slightly above the concentration associated with minimal hemolysis (<8% up to 10 µM). In contrast, the IC_90_ of LL37-1 (25.44 µM) approaches or exceeds concentrations previously associated with detectable hemolytic activity, indicating a reduced safety margin. These comparisons are based on previously reported toxicity assays performed under independent experimental conditions. However, these data suggest a relatively broad selectivity window between antimycobacterial activity and host cell toxicity.

Consistent with the physicochemical properties discussed above, functional assays were performed to determine whether peptide–membrane interactions lead to measurable envelope perturbation. Fluorescence-based membrane integrity assays revealed partial but significant increases in membrane permeability (36–44%) following peptide exposure. Importantly, membrane compromise was not complete, indicating a process of progressive envelope perturbation rather than extensive lysis. These findings are consistent with membrane stress responses rather than direct evidence of pore formation or membrane rupture. This is a hallmark of membrane-active AMPs that induce sublethal stress and metabolic impairment [42,43].

The greater permeability alterations caused by D-LL37, even at lower concentrations than LL37-1, suggest that the D-configuration allows for more prolonged and stable interactions with the *Mtb* lipid envelope. This stability may translate into greater persistence in the bacterial microenvironment, potentially facilitating the insertion and disorganization of the lipid bilayer [44,45].

SEM corroborated the membrane perturbation in the peptide-treated bacilli, which displayed reduced average length. In addition, morphological changes in *Mtb* following exposure to LL-37-derived peptides observed in SEM experiments support the occurrence of structural alterations associated with envelope stress. While some variability in surface appearance and cellular organization was observed in selected images, these features were relatively subtle and variable across observations. In contrast, the most consistent and quantitatively supported effect was a reduction in bacillary length, indicating a measurable morphological response to peptide exposure. These ultrastructural alterations are consistent with envelope perturbation and structural stress rather than complete cellular disintegration.

Studies employing SEM and TEM have reported that synthetic cationic peptides induce membrane wrinkling, surface discontinuities, and altered bacillary morphology without immediate cell lysis [46,47]. Similar structural alterations, including envelope distortion and cellular aggregation, have been observed with other amphipathic α-helical peptides targeting the lipid-rich mycobacterial cell wall [48,49]. Importantly, SEM does not directly demonstrate pore formation or membrane rupture; rather, it reveals surface destabilization and morphological remodeling compatible with membrane stress.

Given the critical role of P-type ATPases in maintaining ionic homeostasis and stress adaptation [22], we examined whether envelope disruption induced by LL-37-derived peptides generated transcriptional responses in these transport systems. Notably, D-LL37 triggered marked upregulation of *ctpF*, *ctpA*, and *ctpH*, whereas LL37-1 produced only modest or non-significant changes. Our findings extend previous observations by linking morphological alterations with the transcriptional activation of P-type ATPases, suggesting that structural changes are accompanied by adaptive ionic stress responses [50].

Our findings suggest that the peptides, by destabilizing the membrane, induce the transcription of the P-type ATPases genes analyzed. However, the study did not include a functional validation that would allow for linking transcriptional changes to physiological consequences. Further experimentation is necessary to determine whether CtpF, CtpE, or CtpF activity is affected, including ATPase activity and metal accumulation assays.

Furthermore, the upregulation of transcription may be related to a general response in mycobacteria, as observed in other studies. Indeed, the expression levels of P-type ATPase genes increased when the bacterium is exposed to toxic substances (sodium hypochlorite, SDS, copper, reactive oxygen and nitrogen species, among others) and under conditions of hypoxia and starvation [22,23,24].

Another limitation of this study is the use of the attenuated *Mtb*H37Ra strain, which differs from virulent strains such as *Mtb*H37Rv in membrane composition and stress-response pathways [13,14]. Therefore, extrapolation of these findings to clinically relevant strains should be made with caution. Nevertheless, P-type ATPases are conserved membrane transporters involved in essential ion homeostasis processes across *Mtb* lineages. Their roles in metal transport, stress adaptation, and virulence have been demonstrated for several members of this family, including CtpF, CtpA, and CtpV [22,23,26].

## 5. Conclusions

In this study, rationally optimized LL-37-derived peptides demonstrated significant antimycobacterial activity against *Mtb*H37Ra. Dose–response analysis revealed that the D-substituted variant (D-LL37) exhibited enhanced potency, with lower IC_50_ and IC_90_ values compared with its L-configured analogue (LL37-1). Structural modifications, including increased cationic charge and stereochemical substitution, were associated with improved inhibitory efficacy; nonetheless, safety must be evaluated before considering clinical use.

Functional assays showed that both peptides induced partial membrane permeabilization under IC_90_ conditions, which ultrastructural alterations observed by SEM corroborated. These findings indicate controlled envelope perturbation rather than extensive bacteriolysis. Furthermore, D-LL37 triggered coordinated transcriptional upregulation of the P-type ATPase genes *ctpF*, *ctpA*, and *ctpH*, linking membrane destabilization with adaptive ion homeostasis responses. Collectively, these results establish a coherent relationship between peptide structural optimization, envelope perturbation, and stress-associated transcriptional activation in *Mtb*. These findings indicate promise, but potential safety limitations require additional study.

## Data Availability

The original contributions presented in this study are included in the article/Supplementary Material. Further inquiries can be directed to the corresponding author.

## Supplementary Materials

The following supporting information can be downloaded at: https://www.mdpi.com/article/10.3390/biom16050665/s1, Figure S1: Original images SEM of Mycobacterium tuberculosis (Mtb) not exposed to antimicrobial peptides (Control C). Figure S2. Original images SEM of Mycobacterium tuberculosis (Mtb) exposed to LL37-1 (P1). Figure S3. Original images SEM of Mycobacterium tuberculosis (Mtb) exposed to D-LL37 (P2).

## References

[1] World Health Organization (2025). Global Tuberculosis Report 2025.

[2] Dheda K., Gumbo T., Maartens G., Dooley K.E., McNerney R., Murray M., Furin J., Nardell E.A., London L., Lessem E. (2017). The Epidemiology, Pathogenesis, Transmission, Diagnosis, and Management of Multidrug-Resistant, Extensively Drug-Resistant, and Incurable Tuberculosis. Lancet Respir. Med..

[3] Wang Y., Cao D., Liu G. (2025). Application of Antimicrobial Drugs in Mycobacterium Tuberculosis and Research Progress. Microb. Pathog..

[4] Mehta K., Spaink H.P., Ottenhoff T.H.M., Van Der Graaf P.H., Van Hasselt J.G.C. (2022). Host-Directed Therapies for Tuberculosis: Quantitative Systems Pharmacology Approaches. Trends Pharmacol. Sci..

[5] Fang R., Jin Y., Kong W., Wang H., Wang S., Li X., Xing J., Zhang Y., Yang X., Song N. (2024). Nano-Strategies Used for Combatting the Scourge of Tuberculosis Infections. Discov. Immun..

[6] Kumar M., Virmani T., Kumar G., Deshmukh R., Sharma A., Duarte S., Brandão P., Fonte P. (2023). Nanocarriers in Tuberculosis Treatment: Challenges and Delivery Strategies. Pharmaceuticals.

[7] Singh P., Khurana H., Yadav S.P., Dhiman K., Singh P., Ashish, Singh R., Sharma D. (2020). Biochemical Characterization of ClpB Protein from Mycobacterium Tuberculosis and Identification of Its Small-Molecule Inhibitors. Int. J. Biol. Macromol..

[8] Chauhan N.K., Anand A., Sharma A., Dhiman K., Gosain T.P., Singh P., Singh P., Khan E., Chattopadhyay G., Kumar A. (2023). Structural and Functional Characterization of Rv0792c from Mycobacterium Tuberculosis: Identifying Small Molecule Inhibitor against HutC Protein. Microbiol. Spectr..

[9] Lupoli T.J., Vaubourgeix J., Burns-Huang K., Gold B. (2018). Targeting the Proteostasis Network for Mycobacterial Drug Discovery. ACS Infect. Dis..

[10] Mookherjee N., Anderson M.A., Haagsman H.P., Davidson D.J. (2020). Antimicrobial Host Defence Peptides: Functions and Clinical Potential. Nat. Rev. Drug Discov..

[11] Magana M., Pushpanathan M., Santos A.L., Leanse L., Fernandez M., Ioannidis A., Giulianotti M.A., Apidianakis Y., Bradfute S., Ferguson A.L. (2020). The Value of Antimicrobial Peptides in the Age of Resistance. Lancet Infect. Dis..

[12] Dwivedi M., Parmar M.D., Mukherjee D., Yadava A., Yadav H., Saini N.P. (2023). Biochemistry, Mechanistic Intricacies, and Therapeutic Potential of Antimicrobial Peptides: An Alternative to Traditional Antibiotics. Curr. Med. Chem..

[13] Daffé M., Marrakchi H. (2019). Unraveling the Structure of the Mycobacterial Envelope. Microbiol. Spectr..

[14] Dulberger C.L., Rubin E.J., Boutte C.C. (2020). The Mycobacterial Cell Envelope—A Moving Target. Nat. Rev. Microbiol..

[15] Ridyard K.E., Overhage J. (2021). The Potential of Human Peptide Ll-37 as an Antimicrobial and Anti-Biofilm Agent. Antibiotics.

[16] Memariani H., Memariani M. (2023). Antibiofilm Properties of Cathelicidin LL-37: An in-Depth Review. World J. Microbiol. Biotechnol..

[17] Ito T., Matsunaga N., Kurashima M., Demizu Y., Misawa T. (2023). Enhancing Chemical Stability through Structural Modification of Antimicrobial Peptides with Non-Proteinogenic Amino Acids. Antibiotics.

[18] Boidin-Wichlacz C., Maresca M., Correia I., Lequin O., Point V., Casanova M., Reinbold A., Iranzo O., Huws S.A., Brodin P. (2025). Potency of All-D Amino Acid Antimicrobial Peptides Derived from the Bovine Rumen Microbiome on Tuberculous and Non-Tuberculous Mycobacteria. Curr. Res. Microb. Sci..

[19] Pinilla G., Coronado Y.T., Chaves G., Muñoz L., Navarrete J., Salazar L.M., Taborda C.P., Muñoz J.E. (2022). In Vitro Antifungal Activity of LL-37 Analogue Peptides against *Candida* spp. J. Fungi.

[20] Alba M.L.S., Durán-Rodriguez A.T., Pulido L.M.S., Escobar-Pérez J., Gutiérrez S.A., Ospina J.N., Bermúdez G.P., Molina L.C.M. (2022). Peptides DLL37-1 and LL37-1, an Alternative to Inhibit Biofilm Formation in Clinical Isolates of Staphylococcus Aureus and Staphylococcus Epidermidis. An. Acad. Bras. Cienc..

[21] Chingaté S., Delgado G., Salazar L.M., Soto C.Y. (2015). The ATPase Activity of the Mycobacterial Plasma Membrane Is Inhibited by the LL37-Analogous Peptide LLAP. Peptides.

[22] Novoa-Aponte L., Soto Ospina C.Y. (2014). *Mycobacterium tuberculosis* P-Type ATPases: Possible Targets for Drug or Vaccine Development. BioMed Res. Int..

[23] Maya-Hoyos M., Mata-Espinosa D., López-Torres M.O., Tovar-Vázquez B., Barrios-Payán J., León-Contreras J.C., Ocampo M., Hernández-Pando R., Soto C.Y. (2022). The ctpF Gene Encoding a Calcium P-Type ATPase of the Plasma Membrane Contributes to Full Virulence of Mycobacterium Tuberculosis. Int. J. Mol. Sci..

[24] López-R M., Maya-Hoyos M., León-Torres A., Cruz-Cacais A., Castillo E., Soto C.Y. (2024). The Copper P-Type ATPase CtpA Is Involved in the Response of Mycobacterium Tuberculosis to Redox Stress. Biochimie.

[25] Hoyos M.M. (2021). ATPasas Tipo P2 Como Blancos Para La Atenuación de Mycobacterium tuberculosis.

[26] López-Ruíz M., Barrios-Payán J., Maya-Hoyos M., Hernández-Pando R., Ocampo M., Soto C.Y., Mata-Espinosa D. (2025). The Plasma Membrane P-Type ATPase CtpA Is Required for Mycobacterium Tuberculosis Virulence in Copper-Activated Macrophages in a Mouse Model of Progressive Tuberculosis. Biomedicines.

[27] Varon H.A., Santos P., Lopez-Vallejo F., Soto C.Y. (2023). Novel Scaffolds Targeting Mycobacterium Tuberculosis Plasma Membrane Ca2+ Transporter CtpF by Structure-Based Strategy. Bioorg. Chem..

[28] Rustad T.R., Roberts D.M., Liao R.P., Sherman D.R. (2008). Isolation of Mycobacterial RNA. Methods Mol. Biol..

[29] Pfaffl M.W., Tichopad A., Prgomet C., Neuvians T.P. (2004). Determination of Stable Housekeeping Genes, Differentially Regulated Target Genes and Sample Integrity: BestKeeper—Excel-Based Tool Using Pair-Wise Correlations. Biotechnol. Lett..

[30] Zhang Z.-T., Wu Y.-C., Dong C.-M. (2025). Cathelicidin LL-37: Mechanisms of Action and Research Progress. Infect. Dis. Res..

[31] Simonetti O., Cirioni O., Goteri G., Lucarini G., Kamysz E., Kamysz W., Orlando F., Rizzetto G., Molinelli E., Morroni G. (2021). Efficacy of Cathelicidin LL-37 in an MRSA Wound Infection Mouse Model. Antibiotics.

[32] Yuan Y., Li J., Wei G., Shen Z., Li B., Wu J., Liu J. (2025). Exploring the Antimicrobial Potential of LL-37 Derivatives: Recent Developments and Challenges. ACS Biomater. Sci. Eng..

[33] Voronko O.E., Khotina V.A., Kashirskikh D.A., Lee A.A., Gasanov V.A.O. (2025). Antimicrobial Peptides of the Cathelicidin Family: Focus on LL-37 and Its Modifications. Int. J. Mol. Sci..

[34] Chen Z., Yu X., Zhang A., Wang F., Xing Y. (2020). De Novo Hydrocarbon-Stapling Design of Single-Turn α-Helical Antimicrobial Peptides. Int. J. Pept. Res. Ther..

[35] Habibie A., Putri R.A., Swasono R.T., Retnaningrum E., Dhar P., Kuczera K., Raharjo T.J., Siahaan T.J. (2025). Improving Conformational Stability and Bacterial Membrane Interactions of Antimicrobial Peptides with Amphipathic Helical Structure. Med. Chem. Res..

[36] Högel P., Götz A., Kuhne F., Ebert M., Stelzer W., Rand K.D., Scharnagl C., Langosch D. (2018). Glycine Perturbs Local and Global Conformational Flexibility of a Transmembrane Helix. Biochemistry.

[37] Yokoo H., Hirano M., Ohoka N., Misawa T., Demizu Y. (2021). Structure–Activity Relationship Study of Amphipathic Antimicrobial Peptides Using Helix-destabilizing Sarcosine. J. Pept. Sci..

[38] Schahl A., Réat V., Malaga W., Birbes C., Czaplicki G., Jolibois F., Yamamoto E., Ramos P., Milon A., Saurel O. (2026). How PGL Finds a Sweet Spot in Phospholipid Membranes: A Combined Multiscale MD and NMR Study. Biophys. J..

[39] Cui S., Guo C., Yan L., He Y., Wu L. (2025). Research on Enhancing Enzymatic Degradation of Anti-Digestive Peptides Containing D-Amino Acids through N-Terminal Acetylation. Bioorg. Chem..

[40] Kuzmin D.V., Emelianova A.A., Kalashnikova M.B., Panteleev P.V., Ovchinnikova T.V. (2017). Effect of N- and C-Terminal Modifications on Cytotoxic Properties of Antimicrobial Peptide Tachyplesin I. Bull. Exp. Biol. Med..

[41] Wątły J., Szarszoń K., Sabieraj M., Kola A., Wieczorek R., Janek T., Valensin D. (2025). Modulating Copper(II) Coordination and Antimicrobial Activity: Effects of D -Amino Acid Substitution and *Retro-Inverso* Modification in Human Saliva MUC7 Peptide. Inorg. Chem..

[42] Nagarajan D., Nagarajan D. (2024). Antimicrobial Peptides. Antibiotics and Their Mechanisms of Action.

[43] Sharma A., Gaur A., Kumar V., Sharma N., Patil S.A., Verma R.K., Singh A.K. (2021). Antimicrobial Activity of Synthetic Antimicrobial Peptides Loaded in Poly-Ɛ-Caprolactone Nanoparticles against Mycobacteria and Their Functional Synergy with Rifampicin. Int. J. Pharm..

[44] Lu J., Xu H., Xia J., Ma J., Xu J., Li Y., Feng J. (2020). D- and Unnatural Amino Acid Substituted Antimicrobial Peptides With Improved Proteolytic Resistance and Their Proteolytic Degradation Characteristics. Front. Microbiol..

[45] Khara J.S., Mojsoska B., Mukherjee D., Langford P.R., Robertson B.D., Jenssen H., Ee P.L.R., Newton S.M. (2020). Ultra-Short Antimicrobial Peptoids Show Propensity for Membrane Activity Against Multi-Drug Resistant Mycobacterium Tuberculosis. Front. Microbiol..

[46] Tan T., Wu D., Li W., Zheng X., Li W., Shan A. (2017). High Specific Selectivity and Membrane-Active Mechanism of Synthetic Cationic Hybrid Antimicrobial Peptides Based on the Peptide FV7. Int. J. Mol. Sci..

[47] Lai C.-W., Lin C.-Y., Tsai M.-C., Chen W.-J., Hsieh C.-C., Lin Z.-J., Shen L.-J., Chen Y.-L., Lai L.-J., Chen S.-H. (2025). From AI to Action: Antimicrobial Peptides Engineered by Generative Adversarial Networks (GANs)-A Novel Approach to Combat Resistant Bacteria. Chem. Eng. J..

[48] Sharma D., Poonam, Shrivastava R., Bisht G.S. (2020). In Vitro Efficacy of Lipid Conjugated Peptidomimetics Against Mycobacterium Smegmatis. Int. J. Pept. Res. Ther..

[49] Casanova M., Maresca M., Poncin I., Point V., Olleik H., Boidin-Wichlacz C., Tasiemski A., Mabrouk K., Cavalier J.-F., Canaan S. (2024). Promising Antibacterial Efficacy of Arenicin Peptides against the Emerging Opportunistic Pathogen Mycobacterium Abscessus. J. Biomed. Sci..

[50] Ricaurte S.N. (2025). Características Microbiológicas Asociadas a La Virulencia de Mutantes Defectivos En Los Transportadores de Membrana CtpF y MmpL7 de Mycobacterium Tuberculosis. https://repositorio.unal.edu.co/items/c5dcd330-3bb3-44a6-bb11-8fef814b5f54.

