# Supplementary material for: Optimized LL-37-Derived Peptides Exhibit Antitubercular Activity, Induce Membrane Disruption, and P-Type ATPase Transcriptional Responses in Mycobacterium tuberculosis
Source: Biomolecules. 2026 Apr 30;16(5):665. doi: 10.3390/biom16050665 (PMC13204550; doi:10.3390/biom16050665)
Supplement: Supplementary file 1 [file biomolecules-16-00665-s001.zip › biomolecules-4241740-supplementary.pdf]

Optimized LL-37–Derived Peptides Exhibit Antitubercular Activity, Induce Membrane Disruption, and P-type ATPase Transcriptional responses in *Mycobacterium tuberculosis*

Paola A. Santos[1,2], Milena Maya-Hoyos[1], Luz Mary Salazar[1], Claudia Andrea Cruz[2], Alver Cruz-Cacais[1], Mayerly Giraldo-Avila[1], Juliana Gómez-Manchego[1], Lineth Valentina Triana[2] and Carlos Y. Soto[1]*

Supplementary Materials

Original Images of Scanning electron microscopy (SEM)

Figure S1. Original images SEM of *Mycobacterium tuberculosis* (Mtb) not exposed to antimicrobial peptides (Control C).

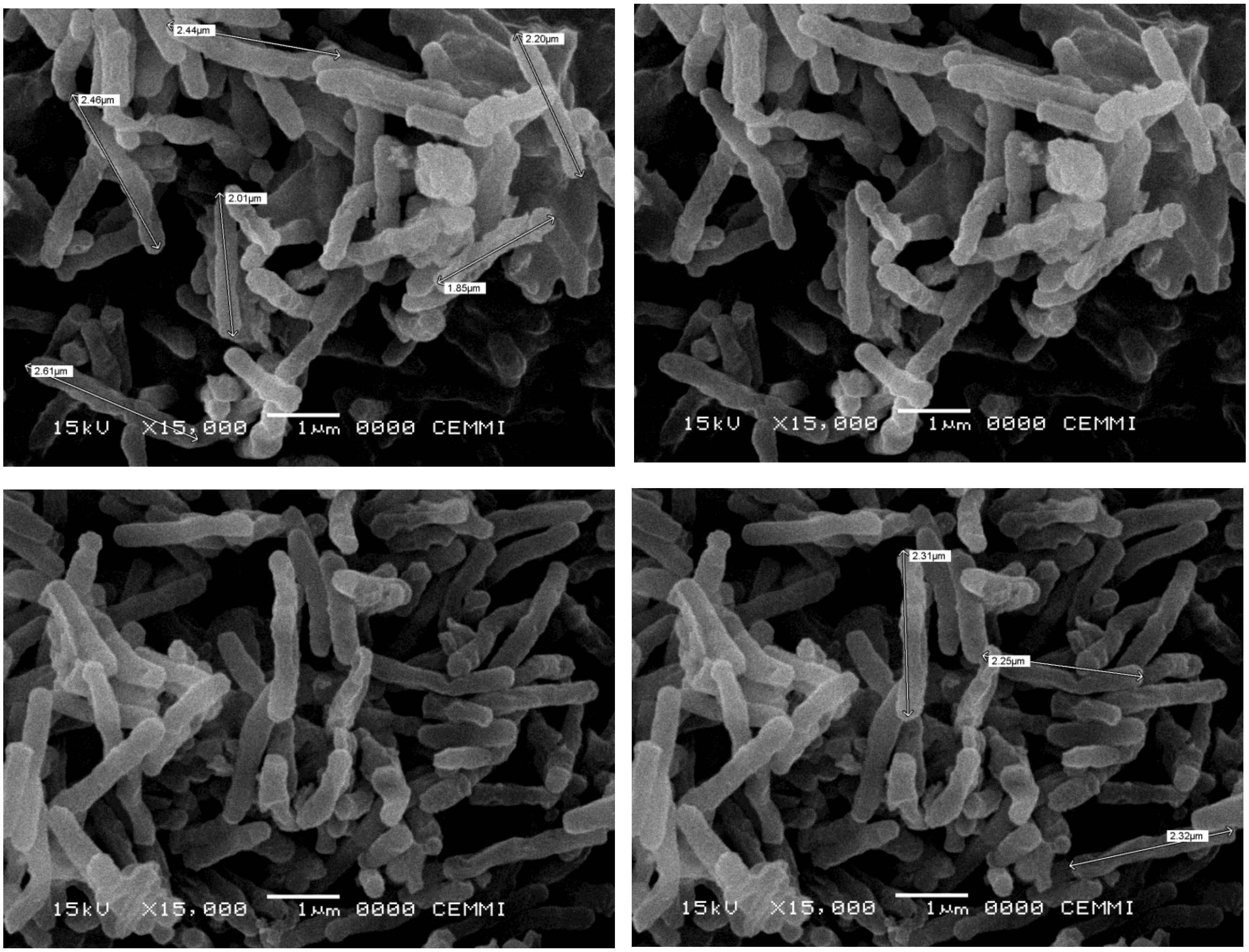

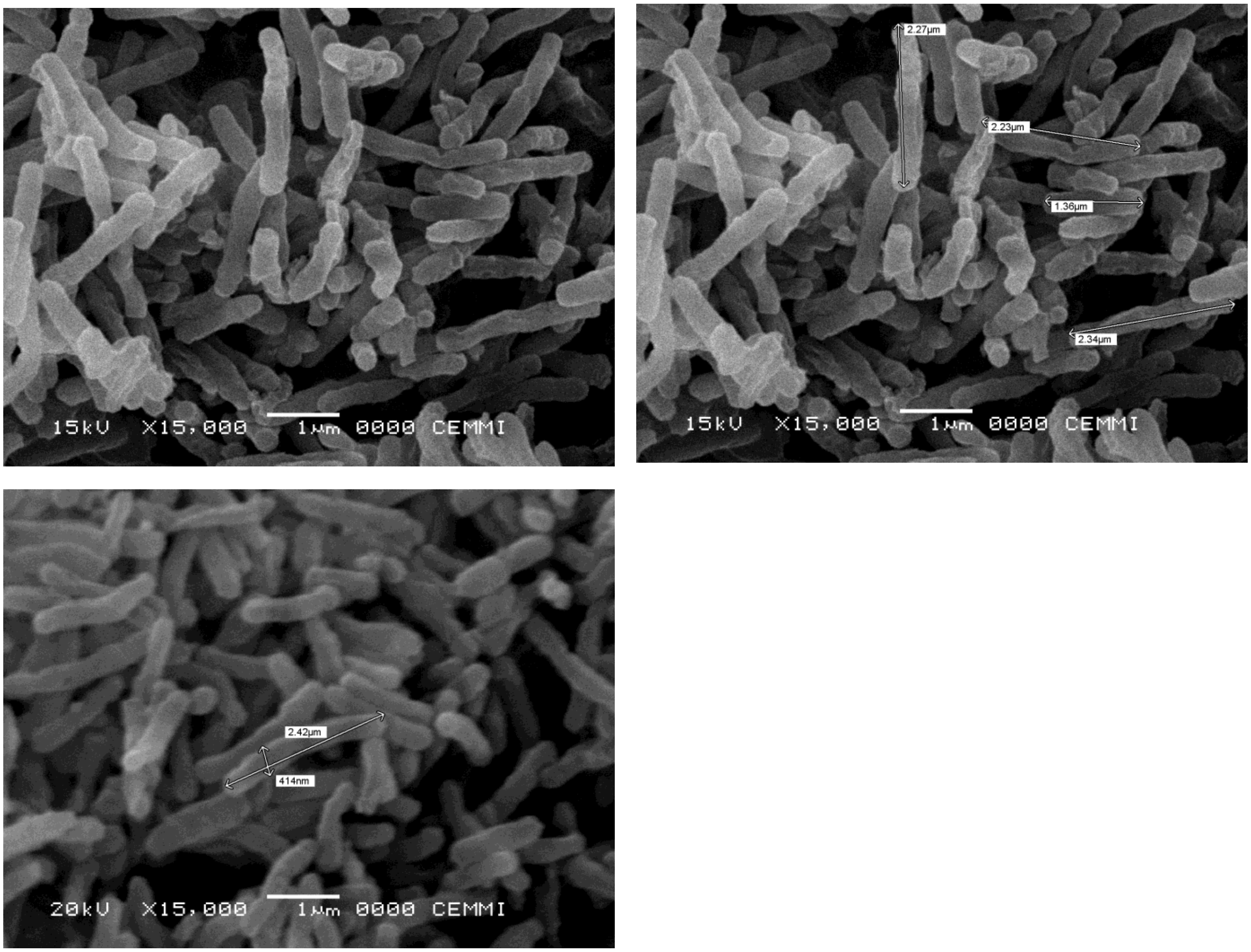

Figure S2. Original images SEM of Mycobacterium tuberculosis (Mtb) exposed to LL37-1 (P1).

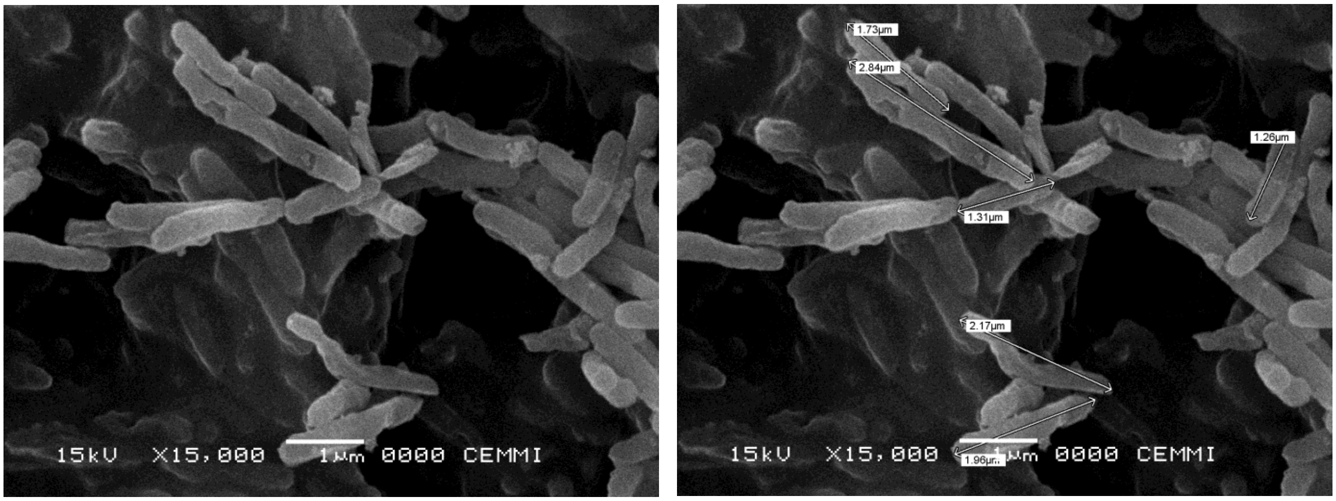

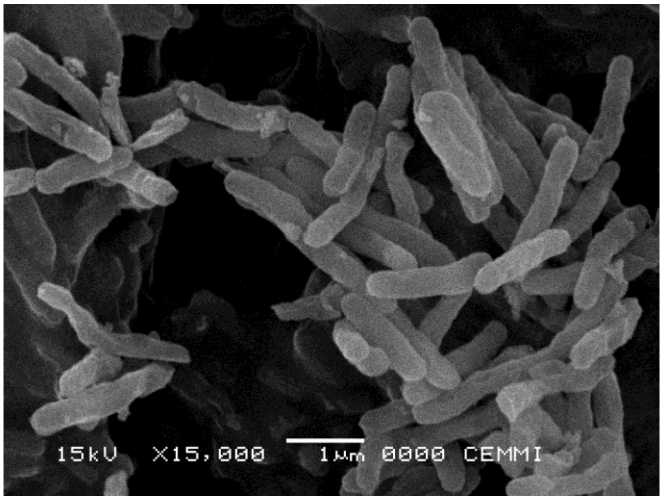
15kV X15,000 1µm 0000 CEMMI

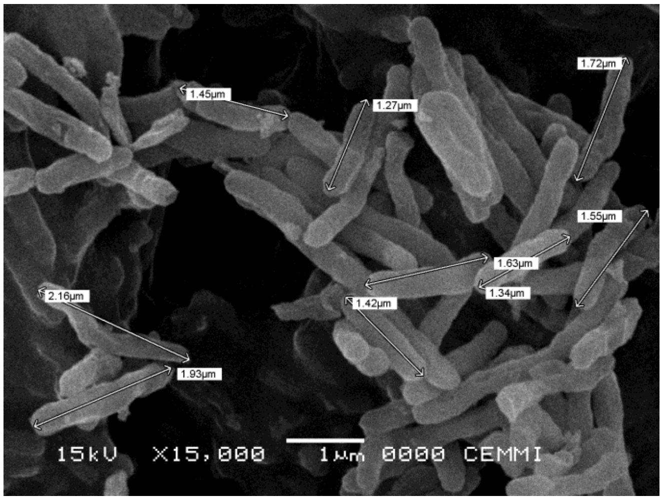
1.45µm
1.27µm
1.72µm
1.55µm
1.63µm
1.34µm
2.16µm
1.42µm
1.93µm
15kV X15,000 1µm 0000 CEMMI

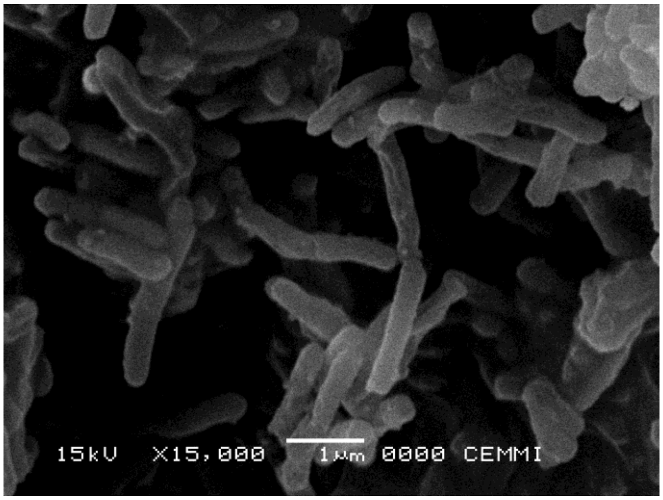
15kV X15,000 1µm 0000 CEMMI

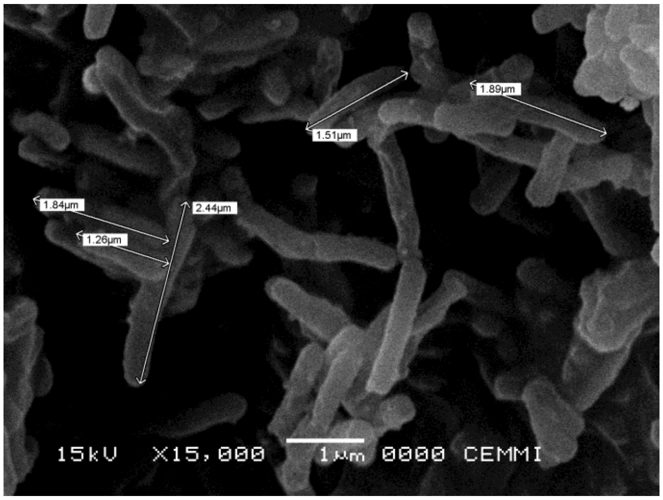
1.89µm
1.51µm
1.84µm
2.44µm
1.26µm
15kV X15,000 1µm 0000 CEMMI

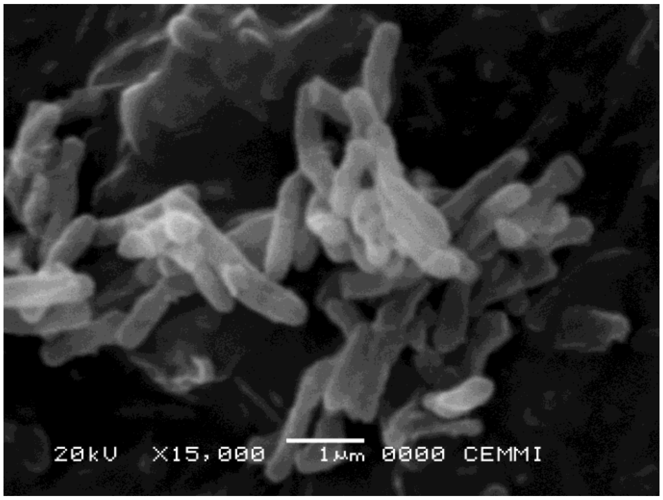
20kV X15,000 1µm 0000 CEMMI

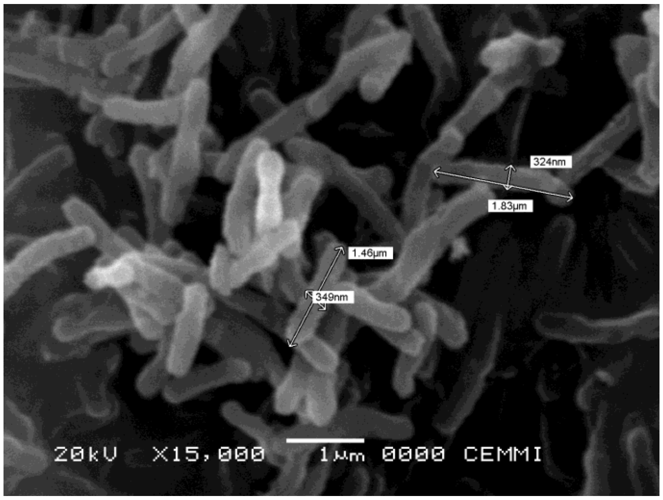
324nm
1.83µm
1.46µm
349nm
20kV X15,000 1µm 0000 CEMMI

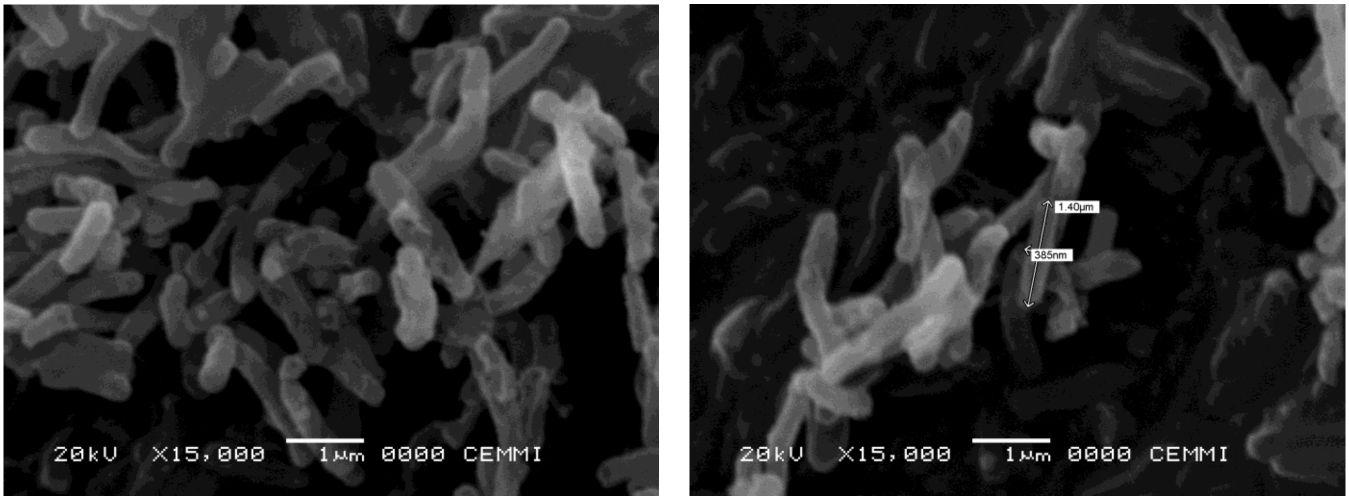

Figure S3. Original images SEM of *Mycobacterium tuberculosis* (Mtb) exposed to D-LL37 (P2).

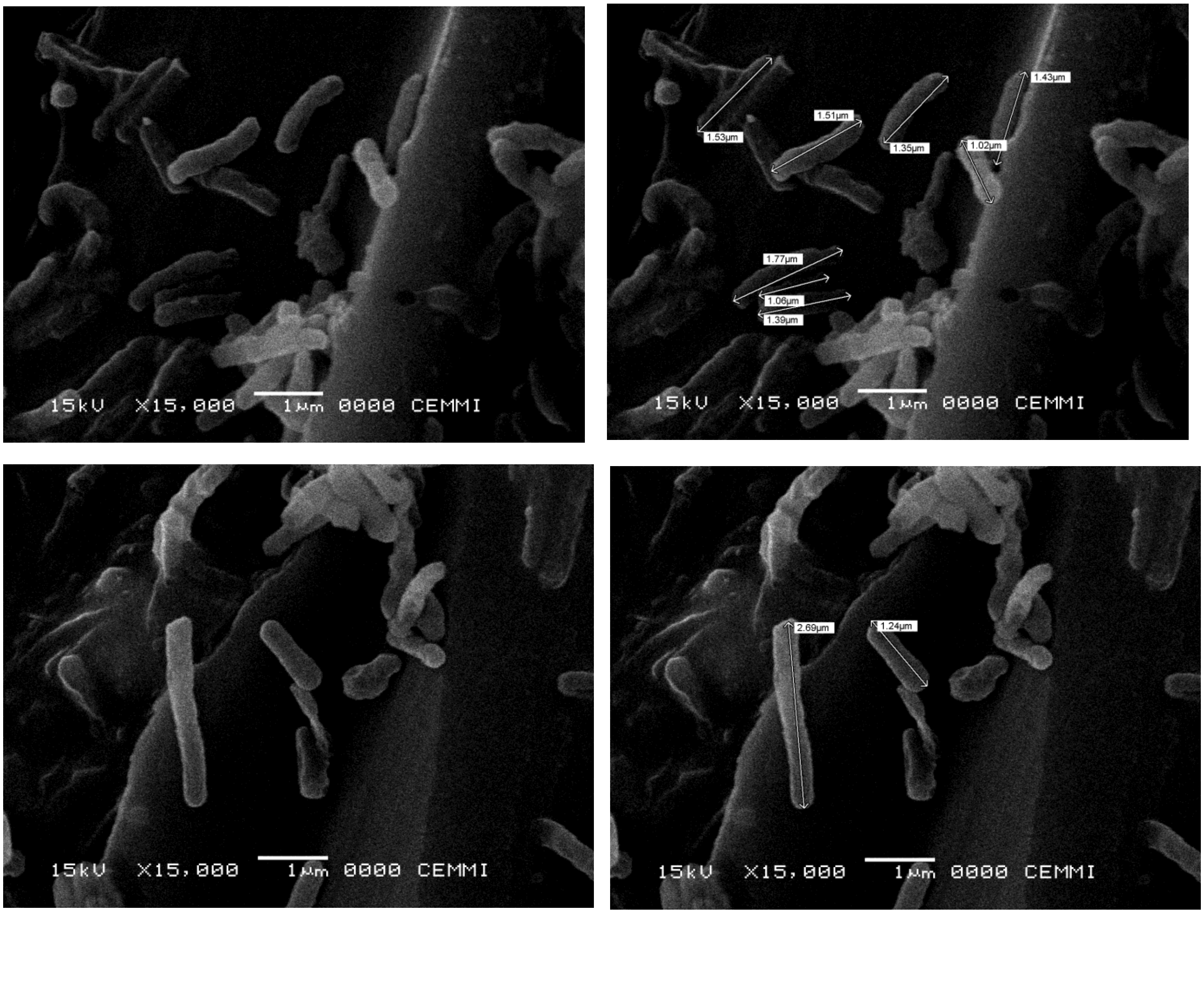

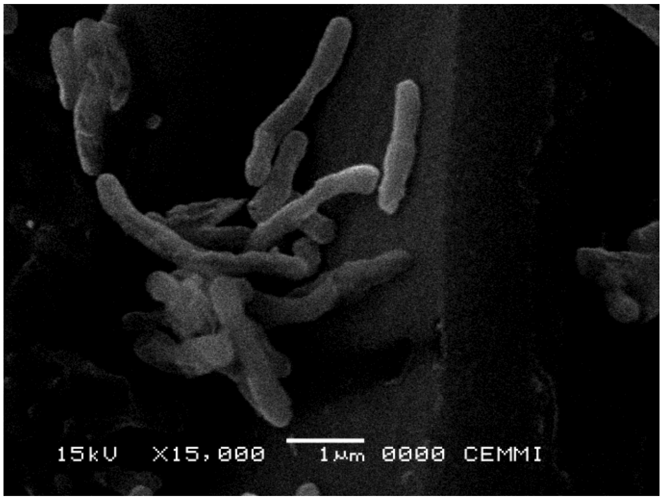
15kV X15,000 1μm 0000 CEMMI

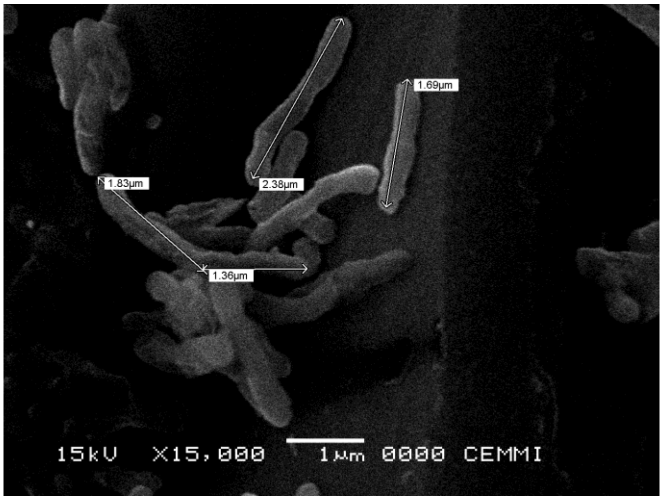
1.69μm
1.83μm
2.38μm
1.36μm
15kV X15,000 1μm 0000 CEMMI

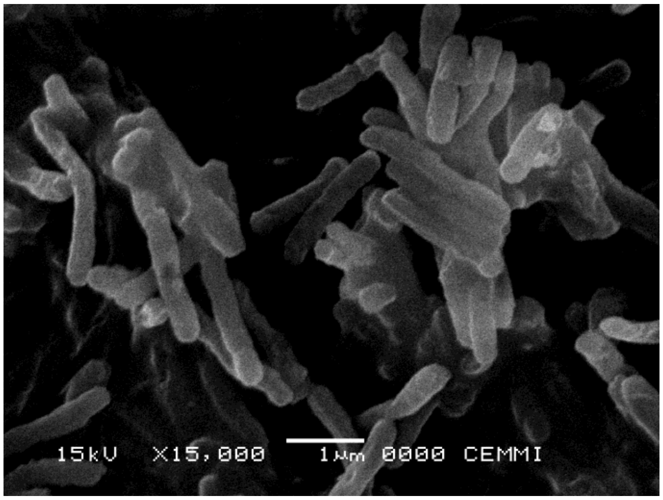
15kV X15,000 1μm 0000 CEMMI

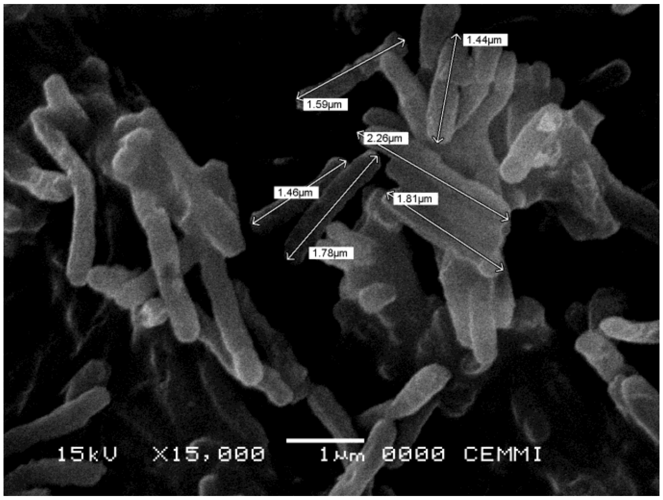
1.44μm
1.59μm
2.26μm
1.46μm
1.81μm
1.78μm
15kV X15,000 1μm 0000 CEMMI

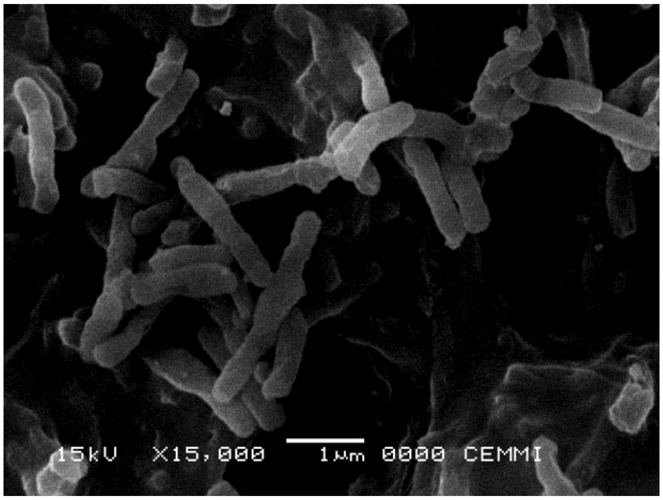
15kV X15,000 1μm 0000 CEMMI

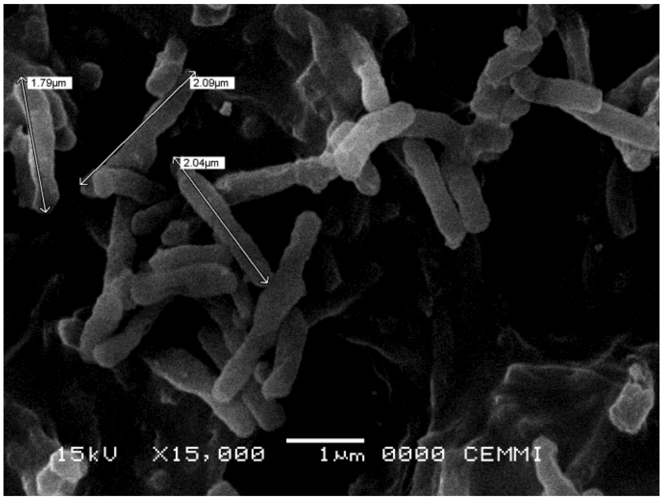
1.79μm
2.09μm
2.04μm
15kV X15,000 1μm 0000 CEMMI

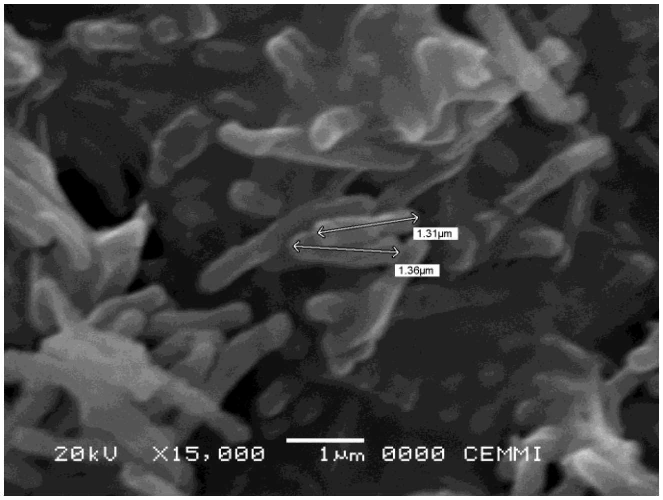
1.31μm
1.36μm
20kV X15,000 1μm 0000 CEMMI

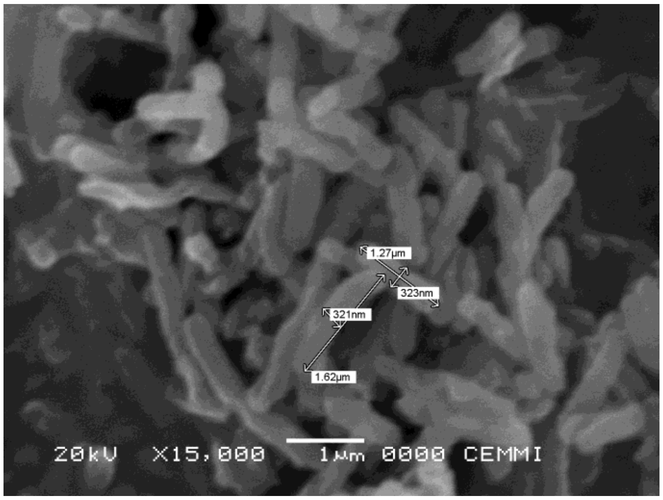
1.27μm
323nm
321nm
1.62μm
20kV X15,000 1μm 0000 CEMMI

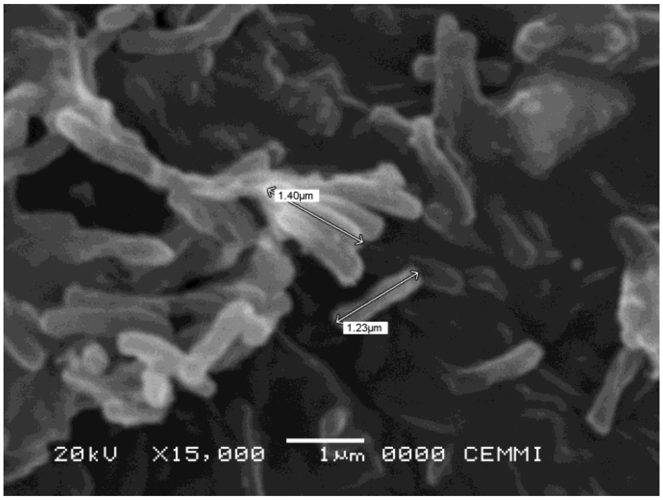
1.40μm
1.23μm
20kV X15,000 1μm 0000 CEMMI

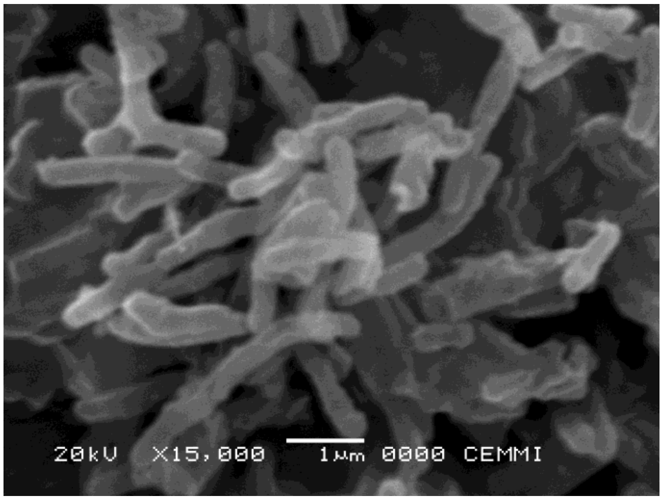
20kV X15,000 1μm 0000 CEMMI
